# Influences of gender in metabolic syndrome and its components among people living with HIV virus using antiretroviral treatment in Hawassa, southern Ethiopia

**DOI:** 10.1186/s13104-016-1953-2

**Published:** 2016-03-05

**Authors:** Agete Tadewos Hirigo, Demo Yemane Tesfaye

**Affiliations:** Department of Medical Laboratory Sciences, Hawassa University College of Medicine and Health Science, P.O. Box 1560, Hawassa, Ethiopia

**Keywords:** Antiretroviral treatment, Gender, Metabolic syndrome, Cardiovascular risks

## Abstract

**Background:**

Data regarding the influences of gender in metabolic syndrome (MetS) among patients using antiretroviral treatment (ART) in Ethiopia is scarce. The aim of this study was to assess the influences of gender in MetS and its components among HIV-infected patients receiving ART.

**Methods:**

A cross-sectional study was conducted between February 2012 and April 2013. Data on demographic, clinical and anthropometric characteristics were collected from 185 HIV patients using ART. Glucose and lipid profiles were measured from overnight fast blood. The International Diabetes Federation (IDF) and United States national cholesterol education program: adult treatment (US NCEP-ATP) panel III criteria were used to define MetS.

**Result:**

A total number of 185 (36.8 % males and 63.2 % females) participants were recruited in this study. The overall prevalence of MetS was 24.3 and 17.8 %, diagnosed using IDF and NCEP-ATP criteria respectively. Using IDF criteria, MetS was significantly higher in females compared to males (33.3 vs. 8.8 %; p = <0.0001) respectively. Low HDL-c and central obesity were significantly higher MetS components in female compared to males (p = 0.003); and (p = <0.0001, using IDF and NCEP-ATP criteria) respectively. BMI >25 kg/m^2^ was significantly associated with MetS in both IDF and NCEP-ATP criteria: unadjusted (UOR) and adjusted odds ratio (AOR) with 95 % CI were 3.0 (1.3–6.5) and 3.8 (1.5–9.8); as well as 3.2 (1.4–7.4) and 3.4 (1.4–7.4) respectively. Furthermore age >40 years was significantly associated with MetS using NCEP-ATP: UOR and AOR (95 % CI) were 3.1 (1.2–8.3), and 3.8 (1–13.70) respectively.

**Conclusion:**

Comprehensive medical care approach including with MetS components are a crucial instruments in order to minimize the risk of developing cardiovascular diseases in HIV-infected patients using ART.

## Background

Metabolic syndrome (MetS) is one of highly prevalent disease, a global challenge at the moment and it has been playing a major role as a marker for metabolic disorders [1, 2]. According to United States National Cholesterol Education Program/Adult Treatment Panel III (US NCEP ATP-III) criteria, MetS is defined by three or more of the following features: abdominal obesity, hypertriglyceridemia, low levels of high density lipoprotein cholesterol (HDL-c), insulin resistance, and hypertension [3]. The characteristics of MetS have been reported to differ between HIV infected patients and the general population, and these differences could be attributed to HIV and/or its associated therapies. The most commonly achieved metabolic criteria for MetS in HIV infected patients were hypertriglyceridemia and low HDL-C, while the least common criterion was increased waist circumference (WC) [4]. Also this disorder confers the risk of developing non-communicable diseases such as cardiovascular diseases and diabetes mellitus among individuals living with HIV infections and being on anti-retroviral treatments (ART) [5]. MetS was commonly and rapidly observed in sub Saharan patients initiating combined ART [6] and also the reports showed that it is more common in women than men [7, 8]. In contrast one study revealed that absence differences in the prevalence of MetS between men and women when stratified by race [9]. MetS diagnosis can be crucial for decision-making in health care regarding cardiovascular disease prevention and patient management. However, data of MetS in HIV-infected subjects in comparison to gender in Ethiopia is scarce.

Therefore the aim of this study was to assess gender influences in the pre-valence of MetS and its components among HIV-infected patients using first line ART.

## Methods

### Study setting and study population

A hospital based prospective cross-sectional study was conducted at the ART clinic of Hawassa University Referral Hospital between February 1, 2013 and April 30, 2013. Hawassa University Referral Hospital is located in Hawassa town, which is the capital city of the southern nations, nationalities and peoples’ region (SNNPR). It is one of the hospitals which provide ART services in the region. During the study period, there were 4504 adult HIV-positive clients in the ART clinic (1796 males and 2708 females). Of whom 2354 were on ART while the remaining 2156 clients were ART naives. Participants used first-line ART regimens that included nucleoside reverse transcriptase inhibitors (NRTIs): lamivudine (3TC), Zidovudine (ZDV), or Stavudine (d4T) or Tenofovir disoproxil fumarate (TDF) with one of non-nucleoside reverse transcriptase inhibitors (NNRTIs): either NVP or EFV. Patients who had had their therapy regimens changed during follow-up were excluded. All participants included were having age ≥18 years and have a good ART adherence (adherence rate ≥95 %).

### Sample size and sampling technique

The sample size was calculated based on single population proportion formula using a confidence interval (CI) of 95 % and a 13 % prior prevalence of MetS among HIV infected patients receiving ART from Benin [6].

$${\text{n}} = (Z{\upalpha_{/2}})^{2} {\text{p}}\,(1-{\text{p}})/{\text{d}}^{2}$$

where, n = sample size, p = proportion of MetS patients who may have MetS and d = assumed marginal error. Based on the above formula and with including 6 % non response rates, the sample size was calculated to be 185. To select participants from the study population, daily patient flow was assessed from the data log at the data clerk office of the ART clinic and patient flow was assessed for a week. Finally the trend showed that the average daily patient flow was approximately 27 cases for ART treated patients. Participants receiving lipid altering therapies, women with pregnancy, known diabetes mellitus patients and renal failures were not included in the study.

### Assessments and measurements

Structured questionnaires were used to collect data on the socio-demographic, clinical and anthropometrics information. Following this, trained ART nurse recorded physical/anthropometric examinations. Blood pressure (BP) was measured by ART clinic nurses using a standard adult arm cuff of mercury type sphygmomanometer after a minimum of 5 min rest of patients in the clinic. Three BP measurements were taken to maintain accuracy and the mean of the three readings was calculated and WC was taken at the midpoint between the lower margin of the last palpable rib and the top of the iliac crest (hip bone). EDTA anticoagulated blood sample was used for measuring of patient’s current CD4^+^ lymphocyte count by using flow-cytometry instrument (Becton–Dickinson, CA, USA). Blood sample was collected from each participant after 8–12 h overnight fast and centrifuged at 3000 cycles/min for 5–10 min, and then serum was obtained for lipid profiles. Furthermore, serum samples were analyzed for fasting glucose level and lipid profile [total cholesterol (TC), HDL-c, LDL-c and TGs] using A25 random access analyzer (BioSystems™, Spain). TC/HDL-c ratio was calculated and enzymatic colorimetric assay method was used for the measurement of TC (CHOD-PAP method) and TGs (GPO-PAP method) while HDL-c and LDL-c measurements were done by utilizing direct homogeneous enzymatic colorimetric assay technique. Glucose level was measured by the glucose oxidase method (GOD-PAP). All the reagents, used for the glucose and lipid profile testing, were from Human Gesellschaft fu^¨^r Biochemica und Diagnostica mBH (Germany).

### Definition of metabolic syndrome

MetS defined according to International Diabetes Federation (IDF), participants had at least three of the following risk factors: abdominal obesity (defined as WC adjusted for Africans is: ≥94 cm for men and ≥80 cm for femeles); raised TG level (≥150 mg/dl); reduced HDL-c (<40 mg/dl in males and <50 mg/dl in females); raised BP (systolic BP ≥ 130 or diastolic BP ≥ 85 mmHg); and raised FBG (≥100 mg/dl) [10]. Whereas according to US NCEP-ATP III definition, abdominal obesity (defined as WC > 102 cm in males and >88 cm in females); raised FBG (≥110 mg/dl) but abnormal lipid profiles and BP levels were alike to IDF criteria [11].

### Statistical analysis

Statistical analysis was done using Statistical Package for Social Sciences (SPSS) Version 20. Categorical variables were summarized as frequencies and percentages, continuous variables were tabulated via mean values and standard deviation while Median values and interquartile range (IQR) were tabulated for skewed variables. Chi square test and fisher exact test were used for categorical variables while comparison of quantitative variables were done by student *t* test or Mann–Whitney *U* test for those parameters did not follow normal distribution. Univariate and multivariate binary logistic regression analysis was used to assess the differences in the distribution of categorical variables for study groups. p < 0.2 was used as a cutoff to include variables for multivariate binary logistic regression model. Finally p < 0.05 at 95 % confidence intervals (CI) was considered as statistically significant.

### Data quality control

Trained nurses working in the ART follow up clinic were concerned in the collection of socio-demographic, clinical and physical/anthropometric characteristics from the patients. Quality control samples were run before running patient samples and along with patient samples in order to check the correct functioning of instruments, laboratory reagents, and technical performances. All laboratory performances were done by lab technologists and using standard operating procedures (SOPs) from sample collection to result releasing.

### Ethical consideration

The study was approved by the Institutional Review Board of the College of Medicine and Health Sciences, Hawassa University. Participation was entirely voluntary, and written consent was obtained from the study participants. Any information obtained during the study was kept with utmost confidentiality. All laboratory analysis was performed free of charge, and the results were provided to the clinicians for possible management.

## Results

### Socio-demographic, clinical and treatment characteristics of study participants

A total number of 185 [68 (36.8 %) males and 117 (63.2 %) females] with a median [interquartile range (IQR)] age of 32 (26.5–38) years, participated in the study. Majority, 54.6, 58.4, and 50.2 percent of the study participants were married, private employed and WHO clinical stage ≥ III respectively. The 22.7, 12.4, and 44.3 percent of HIV patients were drink alcohol (rarely to regularly), had history of cigarette smoking and had sedentary life style respectively. Males were significantly higher in cigarette smoking compared to females (23.5 vs. 6.0 %; p = <0.0001) respectively. Mean [±standard deviation (SD)] of body weight and median (IQR) of CD4+ count were 59.9 (11), and 418 (282-418) respectively. In addition males had significantly higher mean body weight and lower median CD4+ count when compared with females (65 vs. 57; p = <0.0001 and 383 vs. 473; p = 0.005) respectively.

First-line HAART regimens were combinations of 2NRTI and 1NNRTI. All regimens included 3TC. The mean of treatment duration was 43.7 months (range: 6–96 months). The proportion of patients on ZDV/3TC/EFV, and ZDV/3TC/NVP regimens were 35 (18.9 %) and 38 (20.5 %) respectively, while those on d4T/3TC/EFV, and d4T/3TC/NVP regimens were 27 (14.6 %) and 26 (14.1 %) respectively. Those on TDF/3TC/EFV based regimen account for 47 (25.4 %), while the remaining 12 (6.5 %) were on TDF/3TC/NVP. Accordingly, 39.4 % patients were on ZDV; 31.9 % were on TDF; 28.6 % were on d4T; 58.9 % patients were on EFV, and 41.1 % were on NVP (Table 1).

**Table 1:** Characteristics of HIV infected patients using ART with gender category at Hawassa, Southern Ethiopia

Variables	Males (No = 68)	Females (No = 117)
Age (years), median (IQR)	35 (28.2–40.0)	30 (25–35)
<25	8 (11.8)	32 (27.4)
26–35	27 (39.7)	61 (52.1)
36–45	26 (38.2)	21 (17.9)
≥46	7 (10.3)	3 (2.6)
Marital status		
Single	15 (22.0)	24 (20.5)
Married	38 (55.9)	54 (46.1)
Divorced	7 (10.3)	12 (10.2)
Widowed	8 (11.7)	27 (23.1)
Work status		
Unemployed	6 (8.8)	14 (11.9)
Government	16 (23.5)	14 (11.9)
Private	43 (63.2)	53 (25.6)
Student	3 (4.4)	6 (5.1)
A housewife	–	30 (29.0)
Physical exercise		
Sedentary	25 (36.7)	57 (48.7)
Moderate	38 (55.9)	59 (50.4)
Vigorous	5 (7.3)	1 (0.8)
BMI (kg/m^2)^	22.2 (3.2)	22.2 (3.7)
<18.5	4 (5.9)	21 (17.9)
18.5–24.9	50 (73.5)	75 (64.1)
≥25	14 (20.6)	21 (17.9)
CD4, cells/µl, median (IQR)	340 (200–486)	433 (326–572)
<200	14 (20.6)	10 (8.5)
200–400	25 (36.7)	32 (27.4)
≥400	29 (42.7)	75 (64.1)
WHO clinical stage		
I	21 (30.9)	37 (31.6)
II	13 (19.1)	21 (17.9)
III	29 (42.6)	48 (41.0)
IV	5 (7.3)	11 (9.4)
NRTIs		
AZT	21 (30.9)	52 (44.4)
TDF	27 (39.7)	32 (27.3)
d4T	20 (29.4)	33 (28.2)
NNRTIs		
EFV	46 (67.6)	63 (53.8)
NVP	22 (32.3)	54 (46.1)
ART duration (months)	41.4 (19.4)	45.1 (21.5)

Values are the number (percent) for variables unless otherwise indicated

*WHO* world health organization, *NRTI* nucleoside reverse transcriptase inhibitor, *NNRTI* non nucleoside reverse transcriptase inhibitor, *BMI* body mass index, *ART* antiretroviral therapy, *AZT* Zidovudine, *EFV* Efavirenz, *NVP* Nevirapine, *IQR* interquartile range

### The prevalence of MetS and its components

Mean (±SD) of systolic BP, diastolic BP, and WC of the individuals were 112 (15.7), 72.7 (10) and 85.4 (9.7) respectively. The mean HDL-c was significantly lower in male when compared to females (42.1 vs. 51.1; p = <0.0001). However mean TGs, and WC were significantly higher in male compared to females (196 vs. 156; p = 0.005, and 87.7 vs. 85; p = 0.01) respectively. In addition the mean TC/HDL-c ratio and FBG also showed significantly high trend in males (Table 2).

**Table 2:** Gender based prevalence of parameters in HIV infected patients using ART at Hawassa, Southern Ethiopia

Parameters	Males	Females	*p value*
	n = 68	n = 117	
Total cholesterol	188 (49.4)	201 (50.2)	*0.19*
<200 mg/dl, n (%)	43 (63.2)	64 (54.7)	
≥200 mg/dl	25 (36.8)	53 (45.3)	*0.26*
HDL-cholesterol	42.1 (10.3)	51.1 (31.9)	<*0.0001*
LDL-cholesterol	108 (37.7)	115 (42.4)	*0.47*
<130 mg/dl, n (%)	46 (67.6)	82 (70.0)	
≥130 mg/dl, n (%)	22 (32.3)	35 (30.0)	*0.47*
Triglycerides	196 (104.2)	156 (98.5)	*0.005*
FBG	98.6 (15)	92.7 (17)	*0.006*
TC/HDL-c ratio	4.6 (1.3)	4.3 (1.2)	*0.03*
<5, n (%)	42 (61.7)	93 (79.5)	
≥5, n (%)	26 (38.2)	24 (20.5)	*0.009*
WC	87.7 (10)	84 (9)	*0.01*
SBP	113.1 (14.2)	111.3 (16.5)	*0.44*
DBP	73.9 (9.7)	71.9 (10.1)	*0.20*

Values are the Mean ± SD for continuous variables unless and otherwise indicated

*SBP* systolic blood pressure, *DBP* diastolic blood pressure, *TC* total cholesterol, *HDL*-*c* high-density lipoprotein cholesterol, *LDL*-*c* low- density lipoprotein cholesterol, *TG* triglyceride, *FBG* fast blood glucose, *SD* standard deviation, *WC* waist circumference

The prevalence of low HDL-c was significantly higher in males compared to females. In contrast TG ≥ 150 mg/dl was significantly higher in males compared to females. The abnormal WC was significantly higher in female when compared to males (32.5 vs. 5.9 %; p = <0.0001 diagnosed using NCEP-ATP and 70.1 vs. 19.1 %; p = <0.0001 using IDF) respectively. The proportion of MetS was assessed by using two definitions. According to the IDF criteria, the overall prevalence of MetS was 24.3 % (45/185), females have significantly higher rate of MetS, 33.3 % (39/117), when compared to males, 8.8 % (6/68); p = <0.0001. Whereas MetS was 17.8 % in NCEP-ATP criteria, and it was not significantly different between sex (females 17.9 % vs. males 17.6 %; p = 0.95). Based on the IDF criteria 58 (31.3 %) patients had FBG level >100 mg/dl and significantly higher in males compared to females (42.6 vs. 24.8 %; p = 0.01) (Table 3).

**Table 3:** Pattern of MetS and its components in gender among HIV infected patients using ART at Hawassa, southern Ethiopia

Parameters	Males n = 68 (%)	Females n = 117 (%)	p value
SBP ≥130 mmHg	9 (13.2)	13 (11.1)	0.66
DBP >85 mmHg	7 (10.3)	15 (12.8)	0.63
FBG (NCEP-ATP criteria) ≥110 mg/dl	14 (20.6)	13 (11.1)	0.07
FBG (IDF criteria) ≥100 mg/dl	29 (42.6)	29 (24.8)	*0.01*
Hypertensive (≥130/85 mmHg)	7 (10.3)	11 (9.4)	0.84
TG ≥150 mg/dl	39 (57.3)	44 (37.6)	*0.009*
Low HDL-c (<40 mg/dl in males and <50 in females)	39 (57.3)	91 (77.8)	*0.003*
WC (IDF criteria) ≥94 cm in males and ≥80 cm in females	13 (19.1)	82 (70.1)	<*0.0001*
WC (NCEP criteria) ≥102 cm in males and ≥88 cm in females	4 (5.9)	38 (32.5)	<*0.0001*
MetS (IDF criteria)	6 (8.8)	39 (33.3)	<*0.0001*
MetS (NCEP-ATP criteria)	12 (17.6)	21 (17.9)	0.95

### Factors associated with prevalence of metabolic syndrome

Univariate and multivariate analysis models were also applied to assess independent risk factors for MetS. In both models, BMI > 25 kg/m^2^ was significantly associated with MetS in IDF as well as NCEP-ATP III criteria, while being female was significantly associated with MetS in IDF criteria only. Furthermore in NCEP-ATP III criteria, age over 40 years was significantly associated with MetS (Table 4).

**Table 4:** Association of independent factors with metabolic syndrome among HIV infected patients using ART at Hawassa, southern Ethiopia

Explanatory variable	Metabolic syndrome by using IDF criteria			Metabolic syndrome by using NCEP-ATP criteria		
	Presence of MetS = N (%)	COR (95 % CI)	AOR (95 % CI)	Presence of MetS = N (%)	COR (95 % CI)	AOR (95 % CI)
BMI (kg/m^2^)						
≤25	30 (16.2)	1.00	1.00	21 (11.3)	1.00	1.00
>25	15 (8.1)	3.0 (1.3–6.5)	3.8 (1.5–9.8)	12 (6.5)	3.2 (1.4–7.4)	3.4 (1.3–9.2)
p value		*0.006*	*0.005*		*0.006*	*0.01*
Gender						
Male	6 (3.2)	1.00	1.00	12 (6.5)	1.00	1.00
Female	39 (21.1)	5.1 (2.0–12.9)	7.9 (2.6–24.1)	21 (11.3)	1.0 (0.47–2.3)	1.5 (0.24–1.8)
p value		<*0.0001*	<*0.0001*		0.19	0.43
Age (years)						
≤40	41 (22.1)	1.00	1.00	25 (13.5)	1.00	1.00
>40	4 (2.1)	0.66 (0.21–2.0)	2.0 (0.45–9.5)	8 (4.3)	3.1 (1.2–8.3)	3.8 (1.0–13.7)
p value		0.21	0.35		*0.02*	*0.04*
ART use (months)						
≤48	22 (11.9)	1.00	1.00	20 (10.8)	1.00	1.00
>48	23 (12.4)	1.8 (0.95–3.7)	2.3 (1.0–5.4)	13 (7.0)	0.99 (0.46–2.1)	0.8 (0.32–1.9)
p value		0.07	*0.04*		0.20	0.33
Cigarette						
Never smoke	43 (23.2)	1.00	1.00	28 (15.1)	1.00	1.00
Still smoking	2 (1.1)	0.26 (0.06–1.1)	0.17 (0.03–1.0)	5 (2.7)	1.3 (0.45–3.9)	0.47 (0.10–2.1)
p value		0.08	0.05		0.18	0.33
Education						
Illiterate	1 (0.5)	1.00	1.00	3 (1.6)	1.00	1.00
Primary	22 (11.9)	6.7 (0.8–53.3)	10.1 (1.1–94)	15 (8.1)	1.2 (0.31–4.7)	0.85 (0.17–4.3)
p value		0.07	*0.04*		0.16	0.24
Secondary and above	22 (11.9)	6.2 (0.79–49.9)	10.9 (1.1–104)	15 (8.1)	1.1 (0.3–4.4)	1.1 (0.45–2.8)
p value		0.08	*0.04*		0.21	0.37

*ART* antiretroviral therapy, *COR* crude odds ratio, *AOR* adjusted odds ratio, *CI* confidence interval, *BMI* body mass index, *EFV* Efavirenz, *NVP* Nevirapine, *NNRTI* non nucleoside reverse transcriptase inhibitors

## Discussion

The aim of this cross sectional study was to assess the pre-valence of MetS and its components in gender among HIV-infected patients using first line ART in a resource limited setting. The MetS is a complex disturbance represented for a whole of cardiovascular risk factors, usually associated to the central adiposity, dyslipidaemia and insulin resistance.

The overall prevalence of MetS in the study is 24.3 % in IDF where as 17.8 % in NCEP-ATP criteria. In comparable with this finding, a number of international studies point out prevalence’s ranging from 7.4 % to 27 % [12–15]. Conversely high rate of MetS than the present study was reported by Diehl et al. [16] from Londrina, PR (36 %) and Troian et al. from Santa Maria, RS (38.2 %) [17]. Also the report of Benin indicated lower rate (13 %) of MetS compared to our finding [6]. This indicates metabolic complications of HIV infected patients using ART bring in them to future risk of cardiovascular diseases and diabetes, despite improvement of mortality and morbidity conferred by immune reconstitution [18]. Females had significantly higher MetS (33.3 %) compared to males (8.8 %) in IDF criteria. Similarly the studies reported from Latin America and Miami showed that females had significantly high prevalence of MetS compared to males [19, 20]. In addition, this study indicated that female sex was significantly associated risk factor for developing MetS and this in line with the other studies report [21, 22]; in contrast, one study revealed that gender has no significant association with MetS [23]. Among the components of MetS, the abnormal WC was significantly higher in females when compared to males. Similarly the study report from Cameroon indicated that the depicted parameter was significantly higher in females [24]. The present study indicate males have significantly raised TG compared to females, however the study report from Cameroon showed that raised TG was higher in females [24]. The central obesity was significantly higher in females when compared to males in both criteria and it is comparable with the studies report from Brazil [25], and Miami [20]. In addition the Proportion of raised TC/HDL-c ratio was significantly higher in males when compared to females and it indicates a potential risk for HIV-infected patients to develop cardiovascular diseases in a significant proportion in the near future [11, 26–28].

The proportion of reduced HDL-c was significantly higher in females (77.8 %) when compared to males (57.3 %). Comparably the study report from other part Ethiopia showed that females had higher proportion (53.4 %) compared to males (36.7 %); however the rate was lower than the present study [29].

Abnormal BMI was significantly associated with MetS in IDF as well as NCEP-ATP criteria, and likewise several studies report illustrated that the BMI is a quantitative predictor of MetS [30–32]. Furthermore the level of education was directly associated with MetS. Similar findings have also been reported in urban India where the prevalence of MetS was significantly higher among study participants with level of education [33, 34], and Alencastro et al. reported that education to be an independent predictor of MetS [35].

### Limitations of the study

We didn’t do the nutritional assessment due to its difficulty; however the increased risk of cardiovascular diseases associated with described MetS and its components is well known. The other limitations were the cross-sectional nature of the study, small number of male participants, and lack of HIV negative controls. In addition long term use of ART may have an impact on cardiovascular system.

## Conclusion

MetS was detected in 24.3 and 17.8 % in IDF and NCEP-ATP III criteria respectively. According to IDF criteria, the prevalence of MetS was significantly higher compared to males. In both criteria central obesity was significantly higher in females when compared to males. Also MetS components like raised TG was significantly higher in males, where as reduced HDL-c was significantly higher in females. Raised BMI was significantly associated risk factor of MetS in both criteria and female sex was significantly associated with MetS in IDF criteria only. Furthermore age more than 40 year was significantly associated with MetS in NCEP-ATP criteria.

MetS among patients on ART for a longer duration may put them at high risk of cardiovascular diseases. Therefore, screening for fasting blood glucose and lipid profiles should be done before ART initiation and periodically through treatment. Beside physicians/clinicians should be aware of the magnitude of the problem, and ought to include into daily practice interventions to decrease MetS for those patients. Also advising patients for regular exercise and treat with lipid lowering agents for patients with deranged lipid profiles. Furthermore we put forward long term controlled cohort study to evaluate gender and treatment duration influence on MetS among patients on antiretroviral treatment including with nutritional strata.

## Abbreviations

AIDS: acquired immunodeficiency syndrome
HAART: highly active antiretroviral therapy
IDF: international diabetic federation
ZDV: Zidovudine
d4T: Stavudine
EFV: Efavirenz
ART: antiretroviral therapy
HDL-c: HDL-cholesterol
3TC: lamivudine
HIV: human immunodeficiency virus
WHO: World Health Organization
LDL-c: LDL-cholesterol
NNRTIs: non-nucleoside reverse transcriptase inhibitors
NVP: Nevirapine
TC: total cholesterol
TG: triglycerides
SPSS: Statistical package for social sciences
US NCEP-ATP: United States National Cholesterol Education Program, adult treatment panel
MetS: metabolic syndrome
TDF: Tenofovir

## References

[1] Cornier MA, Dabelea D, Hernandez TL, Lindstrom RC, Steig AJ, Stob NR, Van Pelt RE, Wang H, Eckel RH (2008). The metabolic syndrome. Endocr Rev.

[2] Rosolova H, Nussbaumerova B (2011). Cardio-metabolic risk prediction should be superior to cardiovascular risk assessment in primary prevention of cardiovascular diseases. EPMA J.

[3] Expert Panel on Detection Evaluation and Treatment of (2002). High blood cholesterol in adults: third Report of the national cholesterol education program (NCEP) expert panel on detection, evaluation, and treatment of high blood cholesterol in adults (adult treatment panel III) final report. Circulation.

[4] Pao V, Lee GA, Grunfeld C (2008). HIV therapy, metabolic syndrome, and cardiovascular risk. Curr Atheroscler Rep.

[5] Wand H, Calmy A, Carey DL, Samaras K, Carr A, Law MG, Cooper DA, Emery S (2007). Metabolic syndrome, cardiovascular disease and type 2 diabetes mellitus after initiation of antiretroviral therapy in HIV infection. AIDS.

[6] Zannou DM, Denoeud L, Lacombe K, Amoussou-Guenou D, Bashi J, Akakpo J, Gougounon A, Akondé A, Adé G, Houngbé F, Girard PM (2009). Incidence of lipodystrophy and metabolic disorders in patients starting nonnucleoside reverse transcriptase inhibitors in Benin. Antivir Ther.

[7] Schargrodsky H, Hernández-Hernández R, Champagne BM, Silva H, Vinueza R, Silva Ayçaguer LC, Touboul PJ, Boissonnet CP, Escobedo J, Pellegrini F, Macchia A, Wilson E (2008). Assessment of cardiovascular risk in seven Latin American cities. Am J Med.

[8] Sobieszczyk ME, Hoover DR, Anastos K, Mulligan K, Tan T, Shi Q, Gao W, Hyman C, Cohen MH, Cole SR, Plankey MW, Levine AM, Justman J (2008). Prevalence and predictors of metabolic syndrome among HIV-infected and HIV-uninfected women in the womens interagency HIV study. J Acquir Immune Defic Syndr.

[9] Mondy K, Turner OE, Grubb J, Tong S, Seyfried W, Powderly W, Yarasheski K (2007). Metabolic syndrome in HIV-infected patients from an urban, midwestern US outpatient population. Clin Infect Dis.

[10] Alberti KGM, Zimmet P, Shaw J (2006). Metabolic syndrome—a new world-wide definition. A consensus statement from the international diabetes federation. Diabet Med.

[11] Panel on Detection (2002). Evaluation, and treatment of high blood cholesterol in adults (adult treatment panel III) final report. Circulation.

[12] Martin LS, Pasquier E, Roudaut N, Vandhuick O, Vallet S, Bellein V (2008). Metabolic syndrome: a major risk factor for atherosclerosis in HIV-infected patients (SHIVA study). Presse Med.

[13] Hansen BR, Petersen J, Haugaard SB, Madsbad S, Obel N, Suzuki Y, Andersen O (2009). The prevalence of metabolic syndrome in Danish patients with HIV infection: the effect of antiretroviral therapy. HIV Med.

[14] Mondy K, Overton ET, Grubb J, Tong S, Seyfried W, Powderly W, Yarasheski K (2007). Metabolic syndrome in HIV-infected patients from an urban, midwestern US outpatient population. Clin Infect Dis.

[15] Wand H, Calmy A, Carey DL, Samaras K, Carr A, Law MG, Cooper DA, Emery S, Committee ITIC (2007). Metabolic syndrome, cardiovascular disease and type 2 diabetes mellitus after initiation of antiretroviral therapy in HIV infection. AIDS.

[16] Troian MC, Castilhos C, Castilhos M, Bialeski N (2005). Prevalência de síndrome metabólica e dislipidemia em pacientes HIV-positivos em uso de terapia anti-retroviral. J Bras Med.

[17] Diehl LA, Dias JR, Paes ACS, Thomazini MC, Garcia LR, Cinagawa E, Wiechmann SL, Carrilho AJF (2008). Prevalência da Lipodistrofia Associada ao HIV em Pacientes Ambulatoriais Brasileiros: Relação com Síndrome Metabólica e Fatores de Risco Cardiovascular. Arq Bras Endrocrinol Metab.

[18] Samaras K (2007). Metabolic consequences and therapeutic options in Highly active antiretroviral therapy in human immunodeficiency virus-1 infection. J Antimicrob Chemother.

[19] Alvarez C, Salazar R, Galindez J, Rangel F, Castaneda ML, Lopardo G, Cuhna CA, Roldan Y, Sussman O, Gutierrez G, Cure-Bolt N, Seas C, Carcamo C, Castrillo M (2010). Metabolic syndrome in HIV-infected patients receiving antiretroviral therapy in Latin America. Braz J Infect Dis.

[20] Baum M, Rafie C, Lai S, Xue L, Sales S, Page JB, Berkman R, Karas L, Campa A (2006). Coronary heart disease (CHD) risk factors and metabolic syndrome in HIV-positive drug users in Miami. Am J Infect Dis.

[21] Samaras K, Wand H, Law M, Emery S, Cooper D, Carr A (2007). Prevalence of metabolic syndrome in HIV-infected patients receiving highly active antiretroviral therapy using international diabetes foundation and adult treatment panel III criteria. Diabetes Care.

[22] Ivana K, Ariana V, Branka S, Mladen S, Josipa K, Silvije V (2006). Metabolic syndrome in a metapopulation of Croatian island isolates. Croat Med J.

[23] Lauda LG, Mariath AB, Grillo LP (2011). Metabolic syndrome and its components in HIV-infected individuals. Rev Assoc Med Bras.

[24] Dimodi TH, Etame SL, Nguimkeng SB, Mbappe EF, Ndoe EN, Tchinda NJ, Ebene AJ, Ntentié RF, Kingue AB, Angie MM, Paka DG, Kouanfack C, Ngondi LJ, Enyong OJ (2014). Prevalence of metabolic syndrome in HIV-infected Cameroonian patients. World J AIDS.

[25] Lauda LG, Mariath AB, Grillo LP (2011). Metabolic syndrome and its components in HIV-infected individuals. Rev Assoc Med Bras.

[26] Asztalos BF, Schaefer EJ, Horvath KV, Cox CE, Skinner S, Gerrior J, Gorbach SL, Christine Wanke C (2006). Protease inhibitor-based HAART, HDL, and CHD-risk in HIV-infected patients. Atherosclerosis.

[27] Sudano I, Spieker LE, Noll G, Corti R, Weber R, Lüscher TF (2006). Cardiovascular disease in HIV infection. Am Heart J.

[28] Hsia SH, Pan D, Berookim, Lee ML (2006). A population-based, cross-sectional comparison of lipid-related indexes for symptoms of atherosclerotic disease. Am J Cardiol.

[29] Berhane T, Yami A, Alemseged A, Yemane T, Hamza L, Kassim M, Deribe K (2012). Prevalence of lipodystrophy and metabolic syndrome among HIV positive individuals on highly active anti-retroviral treatment in Jimma, south west Ethiopia. Pan Afr Med J.

[30] Jacobson DL, Tang AM, Spiegelman D, Thomas AM, Skinner S, Gorbach SL, Wanke C (2006). Incidence of metabolic syndrome in a cohort of HIV-infected adults and prevalence relative to the US population (National Health and Nutrition Examination Survey). J Acquir Immune Defic Syndr.

[31] Jericó C, Knobel H, Montero M, Ordoñez-Llanos J, Guelar A, Gimeno JL, Saballs P, López-Colomés JL, Pedro-Botet J (2005). Metabolic syndrome among HIV-infected patients:prevalence, characteristics, and related factors. Diabetes Care.

[32] Mondy K, Overton ET, Grubb J, Tong S, Seyfried W, Powderly W, Yarasheski K (2007). Metabolic syndrome in HIV-infected patients from an urban, midwestern US outpatient population. Clin Infect Dis.

[33] Kagaruki G, Kimaro G, Mweya C, Kilale A, Mrisho R, Shao A, Kalinga A, Kahwa A, Ngadaya E, Materu G, Mfinanga S, Mayige M (2015). Prevalence and risk factors of metabolic syndrome among individuals living with HIV and receiving antiretroviral treatment in Tanzania. Br J Med Med Res.

[34] Deedwania PC, Gupta R, Sharma KK, Achari V, Gupta B, Maheshwari A, Gupta A (2014). High prevalence of metabolic syndrome among urban subjects in India: a multisite study. Diabetes Metab Syndr.

[35] Alencastro PR, Fuchs SC, Wolff FH, Ikeda ML, Brandao AB, Barcellos NT (2011). Independent predictors of metabolic syndrome in HIV-infected patients. AIDS Patient Care STDS.

